# Understanding the structural characteristics of macromolecular assemblies in connective tissues.

**DOI:** 10.1063/4.0001070

**Published:** 2025-10-27

**Authors:** Olga Antipova, Joseph Orgel, Thomas Irving, Raul Barrea

**Affiliations:** 1 Argonne National Laboratory, Lemont, IL, USA; 2 Illinois Institute of Technology, Chicago, IL, USA; 3 Illinois Institute ofTechnology, Chicago, IL, USA; 4 DePaul University, Chicago, IL, USA

## Abstract

Synchrotrons provide a variety of X-ray techniques for fast, high-resolution structural, compositional, and functional analysis of biological tissues. Micro- and nano-CT enable ultra-fast high-resolution 3D imaging of relatively large samples, while X-ray fluorescence provides elemental composition of fixed biological samples with highest sensitivity. X-ray fiber diffraction allows to push resolution limit even further and analyze partially-ordered and disordered biological composites, such as bones, muscles, spider silk, and connective tissues. Collagen fibrils are key components of ligaments, tendons, cartilage, intervertebral discs, cardiac valves and chordae tendineae.

Complex architecture, cellular activity, and performance of these tissues strongly depend on proper collagen fiber assembly, governed by proteoglycans. X-ray microdiffraction study of collagenous tissues, such as bone, cardiac tissues, tendons, and cartilage, revealed different fibril distribution patterns and detected presence of different collagen types in these samples. Multimodal x-ray and electron microscopy imaging will shine the light on correlation of collagen fibril structure with its macromolecular ligands and their role in proper tissue functioning, as well as in pathological cases of trauma and disease.

